# Positive and “Enriched” Environments Reverse Traumatic Stress and Reshape Epigenetic Signature of Spermatozoa and Ovulation

**Published:** 2019

**Authors:** Amani Ahmed, Muaweah Ahmad Alsaleh

**Affiliations:** 1- Caen University Hospital, Caen, France; 2- CesamS-CERReV, and INSERM, University of Caen Normandy, Caen, France

In evolutionary theories (Lamarckian and Darwinian), environment and physical changes could be transmitted to the descendants (1). Psychological factors in human beings (2–4) have a negative impact on the germ cells parameters (5). As time goes on, the quality of sperm is deteriorating (5, 6) as well as humanoocyte/egg (7).

A simple change of environment and environmental preconceptional exposure (*i.e*., diet, physical activity, smoking, alcohol consumption, *etc*.) affects the functioning of the genes and the phenotype of the next generation through remodeling epigenetic blueprint of spermatozoa (8, 9). Lifestyle and environmental factors influence the sperm and egg which will affect subsequent generations (10–12). Some studies have examined the transmission of specific behavioral and structural adaptations in relation to the stimulus in the nervous system, from parents to their offspring (10, 13).

Effects of psychological and environmental factors on gene expression persist even after the removal of the inducing agent passed on to subsequent generations (14). So, Genetic Memory can be contemplated in two ways (Biology and Psychology) (10, 15).

Psychological factors which have the impact on fertility are treated in numerous studies on humans (5, 16–18) as psychological stress (7). The life of wars and prisons has an effect on germ cells (Sperm, menstrual irregularity and reproductive function) (7, 19, 20). Men who suffered from anxiety and depression or who have experienced high levels of stress had long-term sperm damage. Stress affects sperm quality over the long term, even slowing down the mental development of off spring. This stress is transmitted from one generation to another and has other perverse effects (21).

Sperm of obese men have a distinct epigenetic signature compared to lean men. The methylome of spermatozoa is dynamically remodeled after weight loss (22). Paternal nutritional status can directly affect the health of offspring (21, 23), suggesting that an epigenetic inheritance phenomenon acquired by the environment is transmitted by gametes (22). Environmental factors as exercise and nutritional status induce acute changes in DNA methylation profiles in human skeletal muscle and adipose tissue (24–28) demonstrating that environmental factors are reshaping the epigenome of somatic tissues (22).

The fertility of humans is impacted by myriad factors (Stress, obesity, alcohol and tobacco, seasonal variations, *etc*.) (28, 30). “Licking” (31), sense of touch and massage can be a great stress reliever, and is very important to emotional health (32), and sexual desire and performance (33). Scientists have found that the process of “Traumatic stress, *etc*.” can be reversed if mice from traumatized lineages are put in “Enriched” environments (34). Psychotherapy could improve psychological and environmental factors and make the environment more positive and richer (35).

Scientists have reached several conclusions by researching humans; the trauma would thus modify the behavior of the traumatized individual but also those of his descendants since one finds a metabolic modification until the third generation. This would mean that trauma also affects germ cells (Spermatozoa and ova) which are the only biological link between generations (34). A positive and nurturing environment help humans withstand the threat (34, 36). The decrease in anxiety (37), improvement of psychological factors (38) with reduced vulnerability to stress factor and shocks (39) are related to bgetting a pregnancy (5).

Negative psychological and environmental factors, war, nutritional status, seasonal variations, physical and social environmental factors and stress have many negative consequences and effects on the germ cells. “Enriched” and positive environments can reverse these factors and have positive consequences on the germ cells in individual that could be transmitted to their off spring.
